# A Magnetic-Balanced Inductive Link for the Simultaneous Uplink Data and Power Telemetry

**DOI:** 10.3390/s17081768

**Published:** 2017-08-02

**Authors:** Chen Gong, Dake Liu, Zhidong Miao, Min Li

**Affiliations:** Institute of Application Specific Instruction-Set Processors, Beijing Institute of Technology, 5 South Zhongguancun Street, Haidian District, Beijing 100081, China; gongchen@bit.edu.cn (C.G.); zhidongmiao@bit.edu.cn (Z.M.); limin2015@bit.edu.cn (M.L.)

**Keywords:** biomedical telemetry, inductive link, simultaneous data and power transmissions, implantable biomedical sensors, intraocular sensors

## Abstract

When using the conventional two-coil inductive link for the simultaneous wireless power and data transmissions in implantable biomedical sensor devices, the strong power carrier could overwhelm the uplink data signal and even saturate the external uplink receiver. To address this problem, we propose a new magnetic-balanced inductive link for our implantable glaucoma treatment device. In this inductive link, an extra coil is specially added for the uplink receiving. The strong power carrier interference is minimized to approach zero by balanced canceling of the magnetic field of the external power coil. The implant coil is shared by the wireless power harvesting and the uplink data transmitting. Two carriers (i.e., 2-MHz power carrier and 500-kHz uplink carrier) are used for the wireless power transmission and the uplink data transmission separately. In the experiments, the prototype of this link achieves as high as 65.72 dB improvement of the signal-to-interference ratio (SIR) compared with the conventional two-coil inductive link. Benefiting from the significant improvement of SIR, the implant transmitter costs only 0.2 mW of power carrying 50 kbps of binary phase shift keying data and gets a bit error rate of 1 × 10${}^{-7}$, even though the coupling coefficient is as low as 0.005. At the same time, 5 mW is delivered to the load with maximum power transfer efficiency of 58.8%. This magnetic-balanced inductive link is useful for small-sized biomedical sensor devices, which require transmitting data and power simultaneously under ultra-weak coupling.

## 1. Introduction

Implantable biomedical sensor systems will play an important role in future medical diagnoses and treatments [1,2]. The biomedical sensor systems tend to require simultaneous wireless power and data transmissions, as the large bulk of the battery always challenges the safety of the implants. The inductive link consisting of a magnetic-coupled coil pair is a viable method to transmit wireless power [3] and data [4]. However, the conventional two-coil inductive link could hardly meet all of the needs of future implantable biomedical sensor devices, as the future devices tend to be miniature and have a deep implantation, which will lead to ultra-weak coupling (typically k=0.01) between the coils. The ultra-weak coupling challenges the conventional inductive link in achieving reliable data transmission under the strong power interference, especially for the uplink (which is transmitted from the external part to the implant part) due to its low transmitted power. Rejecting the power interference and enhancing the uplink signal intensity are key issues when designing the inductive link for the small-sized biomedical sensor devices.

Conventionally, by using load-shift keying (LSK) in the uplink, power and uplink data are simultaneously transmitted through a closely-coupled coil pair for implantable biomedical sensor systems [5,6,7]. The LSK transmits the uplink data by switching the load impedance of the implant coil to induce detectable current variations on the external coil. However, the load switching could substantially affect the wireless power transmission (WPT). The load switching may decrease the power transfer efficiency (PTE) and cause large voltage ripples on the implant load. To reduce the impacts of the load switching on the WPT, some new LSK methods are proposed [8,9]. However, these new LSK methods rely on close coupling (*k* = 0.5∼0.2) and can hardly achieve reliable transmission under weak coupling. To achieve reliable uplink data transmission under weak coupling, a new modulation method was recently proposed in [10], named passive phase shift keying (PPSK). The fundamental aspect of PPSK is similar to LSK. It transmits the uplink data by shorting the implant coil at a specific period to induce larger current variations on the external coil. However, the short connection of the implant coil could also cause larger voltage ripple on the implant load.

To both enhance the reliability under ultra-weak coupling and reduce the impact of the uplink data transmission on WPT, a dual-carrier inductive link scheme is proposed in [11]. In this scheme, dual carriers are used for the power and uplink data transmissions through the same coil pair. It achieves reliable uplink data transmission under ultra-weak coupling without causing significant loss of PTE or ripple on the implant load. However, because the power carrier and the uplink carrier are both transferred through the same coil pair, the external receiver suffers severe power carrier interference. The ratio of signal-to-interference (SIR) could be as low as −75.79 dB. To avoid such severe interference, researchers once proposed to use multiple coil pairs to separate data links from the power link [12,13,14]. They designed a perpendicular structure [15] and an overlapped structure [16] for both the implant part and the external part to reduce the cross-coupling between the power coils and the data coils. However, these methods requires multiple implant coils. The extra implant coil will increase the surface footprint and bulk volume of the implant biomedical sensor devices, which renders the multiple coil-pair method unusable for the biomedical sensor devices on certain areas of the body. To reduce the additional surface footprint of the implant, the figure-eight structure [17] is further proposed. The figure-eight data coils are put inside the power coils. However, it is also unusable for certain areas of the body due to its special structure. For example, these special coil structures cannot be used in our intraocular biomedical sensor device, which will be introduced in the following paragraph, as the figure-eight coil will block the eyesight. Furthermore, this special coil structure is sensitive to coil misalignments.

**Figure 1:**
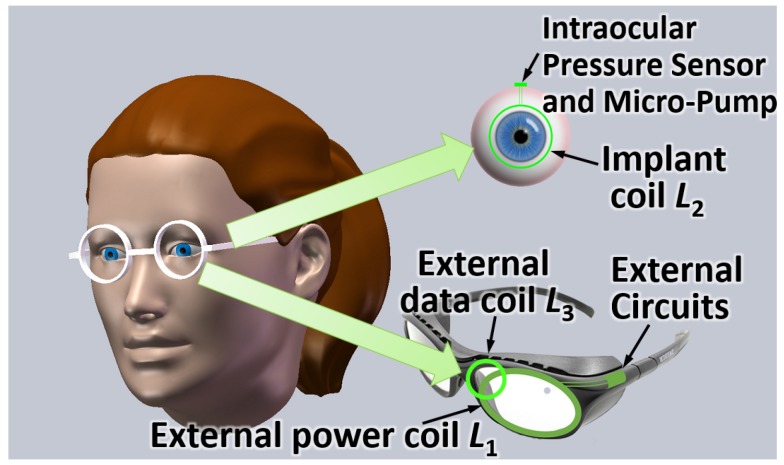
Application scenario of the intraocular sensor system for glaucoma treatment.

In this paper, to address the aforementioned problems, we propose a magnetic-balanced inductive link for our intraocular biomedical sensor device, as seen in Figure 1. The intraocular biomedical sensor device will be implanted in the patient’s eyeball to monitor and regulate the intraocular pressure (IOP) for glaucoma treatment, because lowering IOP is the only evidence-based treatment to prevent the deterioration of glaucoma [18,19]. The uplink sends out the data of the pressure for monitoring the IOP and the data of the peak voltage of the implant load for adaptive power adjustment. When the measured IOP is above the normal IOP, an actuator (micro-pump) is enabled to regulate the IOP. There are three coils (two external coils and one implant coil) used for the wireless power and data transmissions. The external power coil ($L_{1}$) is used for feeding the wireless power, and the external data coil ($L_{3}$) is used for the uplink data receiving, while the implant coil ($L_{2}$) is used for both the wireless power harvesting and the uplink data transmitting. Dual carriers (i.e., power carrier and uplink data carrier) are used for the power and uplink data transmissions, respectively. The two external coils are designed to be partially overlapped and magnetically balanced; thus, the power carrier interference to the uplink receiving can be minimized. The positions of the two external coils are carefully optimized based on the deduced formula of self-inductance and mutual inductance. We further optimize a key resistor of the inductive link circuits to minimize the impacts of the implant uplink transmitter on the WPT. The effects of coil misalignments are also evaluated. Finally, a prototype was implemented and measured. The magnetic-balanced inductive link achieves significant improvement of the SIR compared with the conventional two-coil inductive link. Benefiting from this substantial SIR improvement, the prototype achieves reliable uplink data and power transmissions under ultra-weak coupling with negligible interactions.

The rest of the paper is organized as follows. Section 2 presents a system overview of the magnetic-balanced inductive link. Section 3 presents the equivalent model and analysis of the magnetic-balanced inductive link circuit. Section 4 discusses the coil parameter optimizations, which aim to minimize the power carrier interference and to enhance the received uplink signal intensity. Section 5 discusses a key resistor optimization, which aims to minimize the impacts of the implant uplink transmitter on the WPT. Section 6 evaluates the effects of coil misalignments. Section 7 presents the prototype implementation and the measured results. Section 8 is the Conclusion. Appendix A presents the modeling of self-inductance and mutual inductance of the coils, which are wound using enameled wires.

## 2. Magnetic-Balanced Inductive Link System

### 2.1. System Overview

**Figure 2:**
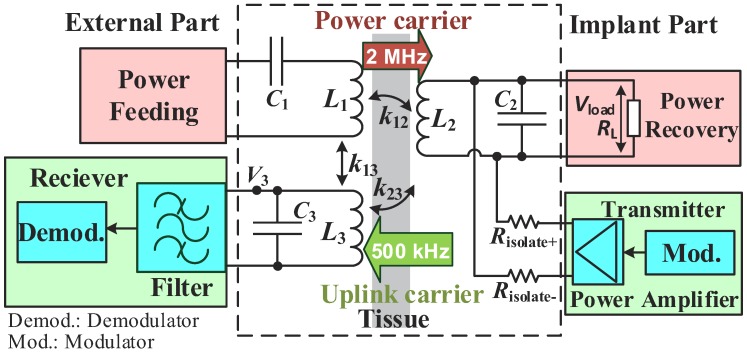
Block diagram of the magnetic-balanced inductive link system.

Figure 2 shows the block diagram of the magnetic-balanced inductive link system. This system is composed of a WPT subsystem (pink blocks), an uplink subsystem (green blocks) and three coils (the external power coil $L_{1}$, the external data coil $L_{3}$ and the implant coil $L_{2}$). The external power coil ($L_{1}$) is used for feeding the wireless power, and the external data coil ($L_{3}$) is used for the uplink data receiving, while the implant coil ($L_{2}$) is used for both the wireless power harvesting and the uplink data transmitting. Dual carriers (i.e., power carrier and uplink data carrier) are used for the power and uplink data transmissions, respectively. The uplink data are modulated on the uplink carrier using binary phase shift keying (BPSK). The power carrier operates at 2 MHz ($f_{p}$) in consideration of less energy loss on biological tissues [20]. The uplink carrier operates at 500 kHz ($f_{\mathrm{ul}}$). It is $\frac{1}{4}$-times lower than $f_{p}$ to leave a sufficient transition band span for filtering out the power carrier interference before the uplink demodulation. By the way, the downlink data (which are transmitted from the external to the implant) could be modulated on the power carrier. As there is no interference problem between the power link and the downlink, the downlink is not discussed in this paper.

As seen in Figure 2, the power feeding unit drives the external power coil ($L_{1}$) to generate the power carrier, and the power recovery unit receives the power carrier from the implant coil ($L_{2}$). At the same time, the implant transmitter drives the same implant coil to transmit the modulated uplink carrier through two isolation resistors ($R_{\mathrm{isolate}+}$ and $R_{\mathrm{isolate}-}$). $R_{\mathrm{isolate}+}$ and $R_{\mathrm{isolate}-}$ are designed to be high impedance to isolate the implant transmitter from the power recovery unit circuits for minimizing the impacts of the uplink data transmission on the WPT. The external receiver acquires the uplink signal from the external data coil ($L_{3}$). The external receiving filter (blue block) further blocks the power carrier and enhances the uplink signal. Then, the clean uplink signal is fed to the uplink demodulator (blue block). Note that $L_{1}$ and $L_{2}$ both resonate at the power carrier frequency ($f_{p}$) with their tuning capacitors $C_{1}$ and $C_{2}$, respectively, in order to derive high PTE. $L_{3}$ resonates at the uplink carrier frequency ($f_{\mathrm{ul}}$) with its tuning capacitor $C_{3}$ to increase the link gain of the uplink.

### 2.2. Magnetically-Balanced Structure

**Figure 3:**
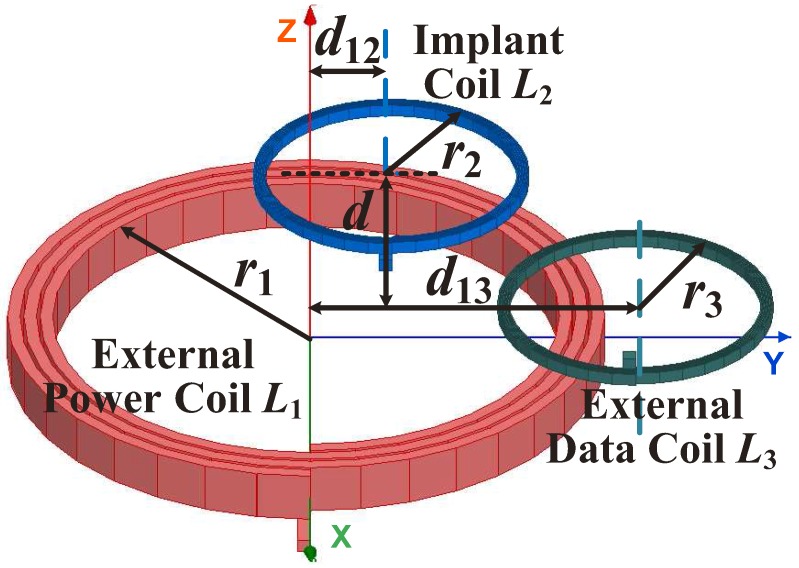
3D model of the magnetic-balanced inductive link.

The positions of the three coils and their notations are demonstrated in Figure 3. The coil separation (*d*) denotes the distance between the external power coil and the implant coil. The distance ($d_{13}$) denotes the center distance between the partially-overlapped external data coil and the external power coil. The skew distance ($d_{12}$) denotes a small offset of the implant coil towards the external data coil along the positive Y axis. Associating Figure 3 with Figure 2, it can be seen that the coupling ($k_{12}$) between $L_{1}$ and $L_{2}$ is used for the WPT. The coupling ($k_{23}$) between $L_{2}$ and $L_{3}$ is used for the uplink data transmission. However, the coupling ($k_{13}$) between $L_{1}$ and $L_{3}$ can transfer the power carrier to $L_{3}$, which induces substantial interference on the uplink signal receiving. To reduce the strong power carrier interference, the two external coils are designed to be partially overlapped and magnetically balanced to minimize their coupling coefficient ($k_{13}$), while $k_{12}$ and $k_{23}$ should be kept at a fairly high level to provide sufficient coupling for the power and uplink data transmissions. The relative distances $d_{13}$ and $d_{12}$ are two significant coil position parameters that need to be optimized and selected in the proposed inductive link, and the optimization procedure is presented in Section 4.

### 2.3. Application Requirements on Coils

In our intraocular biomedical sensor device, the coil sizes are limited by the application requirements, and the size of the implant coil is decided by the doctors from our cooperative hospital. To avoid blocking the eyesight, the implant coil is designed to surround the periphery of the cornea hidden behind the eyelid. The external power coil will be embedded inside the glasses frame in front of and around one eye. Table 1 lists the parameters of the coils we used. The external power coil and the implant coil are optimized for the high PTE, and at the same time, they fulfill the application constraints on the size [21]. The radius of the external data coil is set to be 8 mm without blocking the eyesight. The turn number of the external data coil is optimized for higher intensity of the received uplink signal and at the same it fulfills the system bandwidth requirement. The optimization of the turn number will be introduced in Section 4. The coil separation (*d*) could vary from 15 mm to 40 mm due to the different facial forms and different ways of wearing glasses, which is considered as the operating range of our system.

## 3. Modeling of The Magnetic-Balanced Inductive Link Circuit

### 3.1. Equivalent Modeling

**Figure 4:**
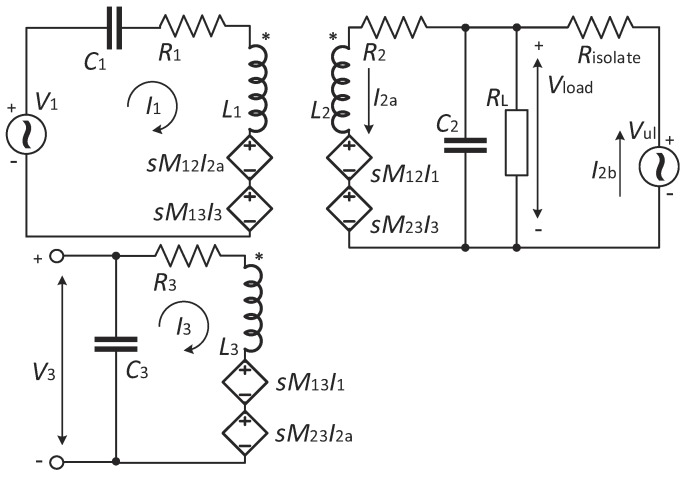
Equivalent circuit model of the magnetic-balanced inductive link circuit.

The equivalent circuit model of the coupling circuit is shown in Figure 4. The power feeding circuit and the uplink transmitter are simplified as ideal AC voltage sources. $V_{1}$ is the ideal AC voltage source of the power carrier. The internal resistance of $V_{1}$ is omitted, because it has negligible direct impact on our inductive link circuit optimization. $V_{\mathrm{ul}}$ is the equivalent AC voltage source of the uplink carrier. $R_{\mathrm{isolate}}$ is the equivalent total resistance of $R_{\mathrm{isolate}+}$ and $R_{\mathrm{isolate}-}$. The internal resistance of $V_{\mathrm{ul}}$ is omitted as it is negligible comparing to $R_{\mathrm{isolate}}$. $L_{1}$, $L_{2}$ and $L_{3}$ are the inductance of the external power coil, the implant coil and the external data coil, respectively. $R_{1}$, $R_{2}$ and $R_{3}$ are the equivalent parasitic resistance of these coils, respectively. $M_{12}$, $M_{23}$ and $M_{13}$ are the corresponding mutual inductance between $L_{1}$, $L_{2}$ and $L_{3}$. $R_{L}$ is the equivalent resistance of the power load, and its value is about 2 kΩ according to our application.

By applying the mesh-current approach on the equivalent circuit model, Equations (1)–(4) are obtained, where *s* is the Laplacian.

(1) $$\begin{array}{cc} V_{1} & =I_{1}\left(sL_{1}+R_{1}+\frac{1}{sC_{1}}\right)+sM_{12}I_{2a}+sM_{13}I_{3}, \end{array}$$

(2) $$\begin{array}{cc} 0 & =sM_{12}I_{1}+I_{2a}\left(sL_{2}+R_{2}\right)+\left(I_{2a}-I_{2b}\right)\frac{R_{L}}{1+sR_{L}C_{2}}+sM_{23}I_{3}, \end{array}$$

(3) $$\begin{array}{cc} V_{\mathrm{ul}} & =I_{2b}R_{\mathrm{isolate}}+\left(I_{2b}-I_{2a}\right)\frac{R_{L}}{1+sR_{L}C_{2}}, \end{array}$$

(4) $$\begin{array}{cc} 0 & =sM_{13}I_{1}+sM_{23}I_{2a}+I_{3}\left(sL_{3}+R_{3}+\frac{1}{sC_{3}}\right). \end{array}$$

Equations (1)–(4) can be expressed in matrix form, as seen in Equation (5), and can be further rewritten in a vector form as seen in Equation (6), where A is the coefficient matrix of the vector I. Thus, the symbolic solution of I can be derived by leveraging Cramer’s rule.

(5) $$\left(\begin{array}{cccc} sL_{1}+R_{1}+\frac{1}{sC_{1}} & sM_{12} & 0 & sM_{13}\\ sM_{12} & sL_{2}+R_{2}+\frac{R_{L}}{1+sR_{L}C_{2}} & -\frac{R_{L}}{1+sR_{L}C_{2}} & sM_{23}\\ 0 & -\frac{R_{L}}{1+sR_{L}C_{2}} & R_{\mathrm{isolate}}+\frac{R_{L}}{1+sR_{L}C_{2}} & 0\\ sM_{13} & sM_{23} & 0 & sL_{3}+R_{3}+\frac{1}{sC_{3}} \end{array}\right)\left(\begin{array}{c} I_{1}\\ I_{2a}\\ I_{2b}\\ I_{3} \end{array}\right)=\left(\begin{array}{c} V_{1}\\ 0\\ V_{\mathrm{ul}}\\ 0 \end{array}\right)$$

(6) AI=V.

### 3.2. Signal-To-Interference Ratio

Since the external receiver acquires the uplink signal from the coil $L_{3}$, the 500-kHz frequency component ($V_{3}\left(j\omega_{\mathrm{ul}}\right)$) of $V_{3}$ is the received uplink signal, and the 2-MHz frequency component ($V_{3}\left(j\omega_{p}\right)$) of $V_{3}$ is the power carrier interference. $\omega_{\mathrm{ul}}$ and $\omega_{p}$ are the angular frequencies of the uplink carrier and the power carrier, respectively. The SIR can be calculated as shown in Equation (7). Equation (7) could be fully expanded by using the symbolic solution of I. However, the full expansion of Equation (7) is omitted, as the fully expansion results in a long and tedious expression.

(7) $$\begin{array}{cc} SIR & =\frac{V_{3}\left(j\omega_{\mathrm{ul}}\right)}{V_{3}\left(j\omega_{p}\right)}=\frac{j\omega_{\mathrm{ul}}M_{13}I_{1}\left(j\omega_{\mathrm{ul}}\right)+j\omega_{\mathrm{ul}}M_{23}I_{2a}\left(j\omega_{\mathrm{ul}}\right)}{j\omega_{p}M_{13}I_{1}\left(j\omega_{p}\right)+j\omega_{p}M_{23}I_{2a}\left(j\omega_{p}\right)}\cdot \frac{1+j\omega_{p}C_{3}R_{3}-{\omega_{p}}^{2}C_{3}L_{3}}{1+j\omega_{\mathrm{ul}}C_{3}R_{3}-{\omega_{\mathrm{ul}}}^{2}C_{3}L_{3}} \end{array}$$

(8) $$\begin{array}{c} \approx \frac{j\omega_{\mathrm{ul}}M_{23}I_{2a}\left(j\omega_{\mathrm{ul}}\right)}{j\omega_{p}M_{13}I_{1}\left(j\omega_{p}\right)}\cdot \frac{1+j\omega_{p}C_{3}R_{3}-{\omega_{p}}^{2}C_{3}L_{3}}{1+j\omega_{\mathrm{ul}}C_{3}R_{3}-{\omega_{\mathrm{ul}}}^{2}C_{3}L_{3}} \end{array}$$

(9) $$\begin{array}{c} =\frac{j\omega_{\mathrm{ul}}k_{23}\sqrt{L_{2}}I_{2a}\left(j\omega_{\mathrm{ul}}\right)}{j\omega_{p}k_{13}\sqrt{L_{1}}I_{1}\left(j\omega_{p}\right)}\cdot \left(j\left(\frac{{\omega_{P}}^{2}}{{\omega_{\mathrm{ul}}}^{2}}-1\right)Q_{3}+\frac{\omega_{p}}{\omega_{\mathrm{ul}}}\right). \end{array}$$

Instead of fully expanding Equation (7), we explore simplifying the SIR equation and then perform a qualitative analysis to guide the optimizations of the coil parameters. As $L_{1}$ is the transmitter coil of the power carrier and $L_{2}$ is the transmitter coil of the uplink carrier, $M_{13}I_{1}\left(j\omega_{p}\right)$ is generally much larger than $M_{23}I_{2}\left(j\omega_{p}\right)$, and $M_{23}I_{2}\left(j\omega_{\mathrm{ul}}\right)$ is much larger than $M_{13}I_{1}\left(j\omega_{\mathrm{ul}}\right)$. Thus, Equation (7) could be approximately simplified to Equation (8). Since $L_{3}$ resonates at the uplink carrier frequency ($f_{\mathrm{ul}}$) with $C_{3}$, we could derive $L_{3}C_{3}=1/{\omega_{\mathrm{ul}}}^{2}$. It is also known that the quality factor of the external data coil can be calculated by $Q_{3}=\omega_{\mathrm{ul}}L_{3}/R_{3}$, and the mutual inductances ($M_{13}$ and $M_{23}$) can be calculated by $M_{13}=k_{13}\sqrt{L_{1}L_{3}}$ and $M_{23}=k_{23}\sqrt{L_{2}L_{3}}$. Therefore, Equation (8) can be rewritten as Equation (9). According to the left part of Equation (9), the uplink SIR could be improved by minimizing the coupling coefficient ($k_{13}$) and increasing the coupling coefficient ($k_{23}$), as $k_{13}$ is the denominator and $k_{23}$ is the numerator. According to the right part of Equation (9), the uplink SIR could be further improved by increasing the quality factor ($Q_{3}$).

## 4. Optimizations of the Coil Position Parameters

In this section, we optimize the distance ($d_{13}$) between the two external coils to improve the SIR. We select proper skew distance $d_{12}$ of the implant coil to avoid the substantial decrease of $k_{23}$ when the coil separation (*d*) is small. In addition, we optimize the turn number ($N_{3}$) of the external data coil to further improve the SIR. In order to guide the optimizations of the coil parameters, the formulas of mutual inductance, self-inductance and coupling coefficient are derived in Appendix A.

### 4.1. Optimization of $d_{13}$ for Minimizing $k_{13}$

**Figure 5:**
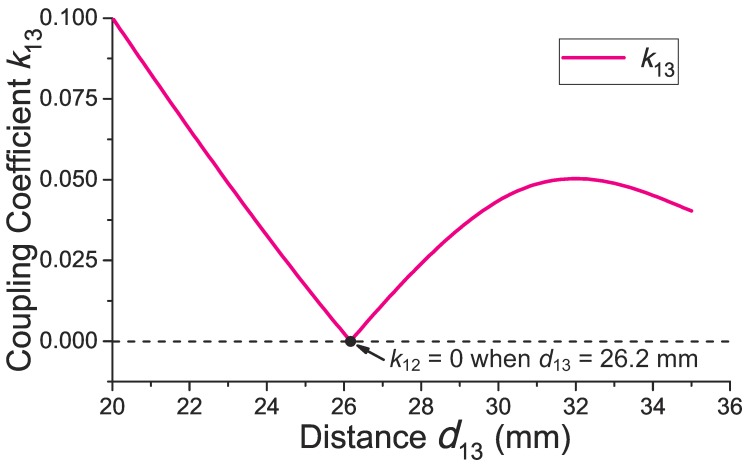
Calculated coupling coefficient ($k_{13}$) versus different $d_{13}$. There is an optimal $d_{13}$ where $k_{13}$ is minimized to approach zero, due to the balanced inside and outside magnetic flux from the external power coil.

When the distance ($d_{13}$) between the external power coil and the external data coil changes, the magnetic flux through the external data coil could be zero at a specific point because of the balanced inside and outside magnetic flux. Thus, the coupling coefficient ($k_{13}$) between the external power coil and the external data coil could be minimized to approach zero at an optimal $d_{13}$. Figure 5 shows the calculated $k_{13}$ versus different $d_{13}$ based on Equation (A17). As seen, there is an optimal $d_{13}=26.2$ mm where $k_{13}$ equals zero. It is known that the direction of the magnetic field, which comes from the inside of the external power coil, is opposite the direction of the magnetic field that comes from the outside of the external power coil. When $d_{13}=26.2$ mm, the magnetic field in the data coil is balanced; the flux from the coil inside magnetic field is equal to the flux from the coil outside magnetic field. Thus, the total flux inside the data coil tends to be zero on this occasion, and the coupling between the coils approaches zero.

### 4.2. Selection of $d_{12}$ for Avoiding a Significant Decrease of $k_{23}$

**Figure 6:**
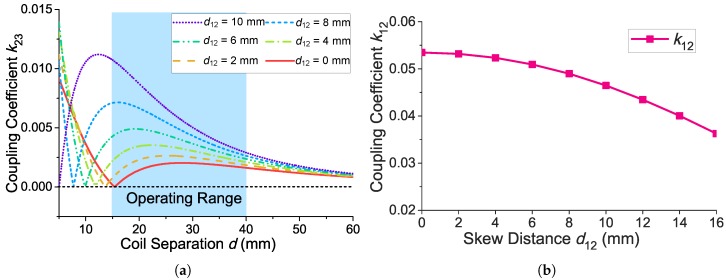
(**a**) Calculated coupling coefficient $k_{23}$ as a function of *d* versus different $d_{12}$; (**b**) calculated coupling coefficient $k_{12}$ versus different $d_{12}$ when the coil separation *d* is 20 mm.

The skew distance $d_{12}$ denotes a small offset of the implant coil towards the external data coil along the positive direction of the Y axis as shown in Figure 3. Figure 6a shows the coupling coefficient ($k_{23}$) as a function of the coil separation (*d*) versus different skew distance ($d_{12}$). In the conventional two-coil inductive power link, the implant coil is coaxial with the external power coil (which means $d_{12}=0$) to maximize the coupling coefficient ($k_{12}$). However, if $d_{12}=0$, $k_{23}$ could decrease significantly and even approach zero when the coil separation (*d*) is small as seen for the red line ($d_{12}=0$ mm) in Figure 6a, because there is also a magnetically-balanced point between the implant coil and the external data coil. We need to avoid this significant decrease of $k_{23}$ in our operating range to keep the uplink data transmission reliable. We thus shift the implant coil towards the external data coil for a short distance ($d_{12}=8$ mm in our case). The selection of $d_{12}=10$ mm can benefit deriving larger $k_{23}$. However, the improvement of $k_{23}$ is remarkable only when the coil separation is small. Along with the increase of the coil separation, the values of $k_{23}$ for different $d_{12}$ tends to be almost the same. When the coil separation is 40 mm, the selection of $d_{12}=10$ mm increases only 9.4% of $k_{23}$ compared with the $k_{23}$ when $d_{12}=8$ mm. Furthermore, the offset of the implant coil could also affect $k_{12}$, while $k_{12}$ is a key parameter to the PTE. Figure 6b shows $k_{12}$ versus different $d_{12}$. As it shows, the increase of $d_{12}$ causes the decrease of $k_{12}$. Therefore, we select $d_{12}=8$ mm instead of $d_{12}=10$ mm to cause less impact on the WPT.

### 4.3. Optimization of Coil Turn Number

A higher quality factor ($Q_{3}$) of the external data coil can further improve the SIR as discussed in Section 3.2. Higher $Q_{3}$ could be obtained by more turns of the external data coil. However, higher $Q_{3}$ also induces a narrower bandwidth ($BW=f_{\mathrm{ul}}/Q_{3}$) of the uplink. Therefore, the turn number of the external data coil should be optimized to have a higher quality factor, but at the same time satisfy the requirement of the bandwidth.

**Figure 7:**
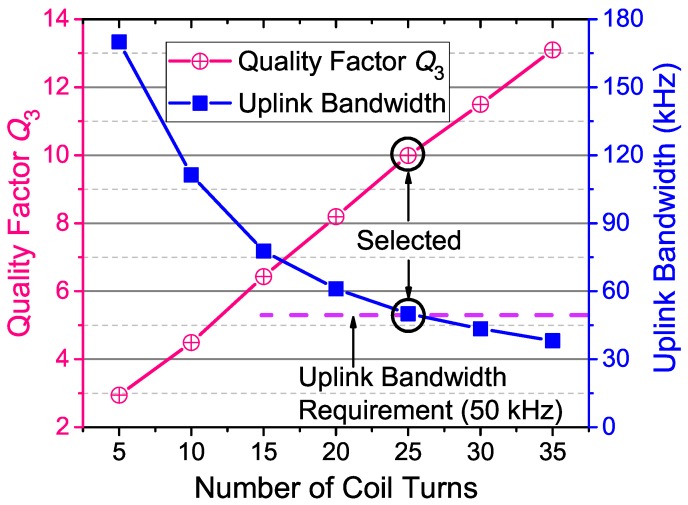
Measured quality factor of the external data coil and its bandwidth on the 500-kHz carrier versus the number of coil turns.

We manufactured a set of external data coils with different numbers of turns. Figure 7 shows the measured quality factor and its bandwidth. The quality factors are measured by using a network analyzer (E5071, Keysignt, Santa Rosa, CA, USA) at the uplink carrier frequency ($f_{\mathrm{ul}}\;=\;500$ kHz). It can be seen that more turns of the coil contributes to higher quality factor. The bandwidth requirement for our intraocular sensor system is BW=50 kHz to achieve the maximum 50 kbps data rate. Thus, the specified target $Q_{3}$ for the external data coil is $Q_{3}=f_{\mathrm{ul}}/BW=10$. To derive the target $Q_{3}$, the number of turns is selected to be 25 according to Figure 7.

## 5. Optimization of the Isolation Resistance $R_{\mathrm{isolate}}$

The resistance of $R_{\mathrm{isolate}}$ is a key circuit parameter, which needs to be optimized in this magnetically-balanced inductive link circuit. The uplink transmitter drives the implant coil though $R_{\mathrm{isolate}}$. $R_{\mathrm{isolate}}$ determines the transmitted power of the uplink transmitter. Furthermore, $R_{\mathrm{isolate}}$ could affect the loaded quality factor of the implant coil $L_{2}$ as $R_{\mathrm{isolate}}$ is directly connected to the implant coil $L_{2}$ in parallel. The loaded quality factor is a key factor to the PTE, and a higher loaded quality factor benefits higher power transfer efficiency [22]. The loaded quality factor $Q_{L\_\mathrm{Conventional}}$ of the conventional inductive link can be found from [23], as Equation (10) shows. Similarly, the loaded quality factor $Q_{L\_\mathrm{Proposed}}$ of our proposed inductive is shown in Equation (11). In order to reduce the impact of the uplink data transmission on the WPT while keeping the uplink signal intensity satisfied, the selection of $R_{\mathrm{isolate}}$ is a trade-off between the loaded quality factor and the received uplink signal power.

(10) $$Q_{L\_\mathrm{Conventional}}=\frac{1}{\frac{R_{2}}{\omega_{p}L_{2}}+\frac{\omega_{p}L_{2}}{R_{L}}}=\frac{1}{R_{2}\sqrt{\frac{C_{2}}{L_{2}}}+\frac{1}{R_{L}}\sqrt{\frac{L_{2}}{C_{2}}}}$$

(11) $$Q_{L\_\mathrm{Proposed}}=\frac{1}{\frac{R_{2}}{\omega_{p}L_{2}}+\frac{\omega_{p}L_{2}}{R_{L}}+\frac{\omega_{p}L_{2}}{R_{\mathrm{isolate}}}}=\frac{1}{R_{2}\sqrt{\frac{C_{2}}{L_{2}}}+\left(\frac{1}{R_{L}}+\frac{1}{R_{\mathrm{isolate}}}\right)\sqrt{\frac{L_{2}}{C_{2}}}}$$

**Figure 8:**
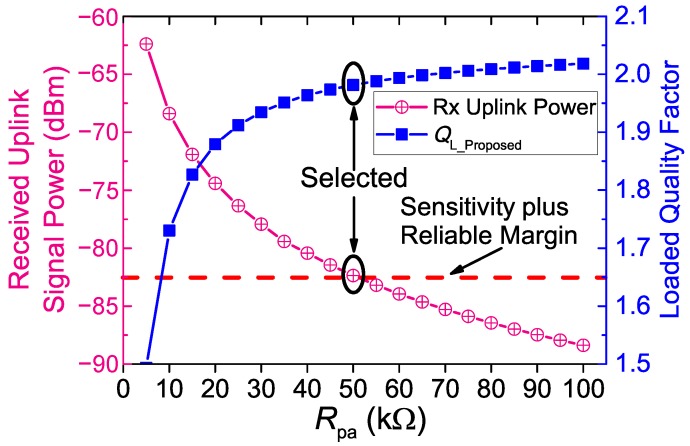
Simulated received uplink signal power and the loaded quality factor $Q_{L\_\mathrm{Proposed}}$ versus $R_{\mathrm{isolate}}$, when the coil separation *d* = 40 mm.

Figure 8 shows the calculated loaded quality factor $Q_{L\_\mathrm{Proposed}}$ and the Pspice simulated received uplink signal power versus $R_{\mathrm{isolate}}$ at the maximum coil separation (*d* = 40 mm) in our operating range. It shows that higher resistance of $R_{\mathrm{isolate}}$ leads to a higher loaded quality factor, but lower received uplink signal power. Figure 8 also shows the uplink receiver sensitivity, which is the minimal required signal power to ensure reliable receiving. The sensitivity is defined in Equation (12), where noise is the thermal noise floor (−174 dBm/Hz); BW is the bandwidth (50 kHz); SNR is the required signal-to-noise ratio (11.5 dB for $1\times 10^{-7}$ error probability of BPSK demodulation); NF is the noise figure (about 11 dB in our prototype); Loss is the implementation loss (about 12 dB in our prototype). Thus, the sensitivity of our uplink receiver is −174+47+11.5+11+12=−92.5 dBm. The minimum uplink received power required is set to be −82.5 dBm, including a 10-dB reliable margin. When the coil separation is maximal (40 mm), the received uplink signal power is minimal. If the minimal power of received uplink signal in the operating range is higher than the receiver sensitivity, it can be ensured that the uplink is reliable in the entire operating range. As Figure 8 shows, when $R_{\mathrm{isolate}}$ is larger than 50 kΩ, the minimal received uplink signal power will be lower than the sensitivity plus the reliable margin. Therefore, $R_{\mathrm{isolate}}$ is selected to be 50 kΩ to achieve a higher loaded quality factor while at the same time fulfilling the constraint of the receiver sensitivity.

(12) $$\mathrm{Sensitivity}=\mathrm{Noise}+10\log\left(\mathrm{BW}\right)+\mathrm{SNR}+\mathrm{NF}+\mathrm{Loss}.$$

Through calculating and comparing $Q_{L\_\mathrm{Proposed}}=1.98$ and $Q_{L\_\mathrm{Conventional}}=2.06$, the decrease of the loaded quality factor caused by the isolation resistors is only 3.9% when $R_{\mathrm{isolate}}=50$ kΩ. In addition, $R_{\mathrm{isolate}}$ dominates the load impedance of the implant transmitter. The transmitted power of the uplink thus can be derived as Equation (13), where $V_{\mathrm{ul}}$ = 3.3 V is the output voltage root-mean-square (RMS) of the uplink transmitter for our device. When $R_{\mathrm{isolate}}=50$ kΩ, the uplink transmitted power is only ∼0.2 mW, which is acceptable for most implantable biomedical sensor devices.

(13) $$P_{\mathrm{Tx}\_\mathrm{ul}}\approx \frac{{V_{\mathrm{ul}}}^{2}}{R_{\mathrm{isolate}}}=0.2\;\mathrm{mW}$$

## 6. Effects of Coil Misalignments

The evaluation of coil misalignment effects is important in the design of inductive links for implantable sensor devices. In our intraocular sensor device, the implant coil could also be moved due to the movement of the eyeball and the displacement of the glasses.

**Figure 9:**
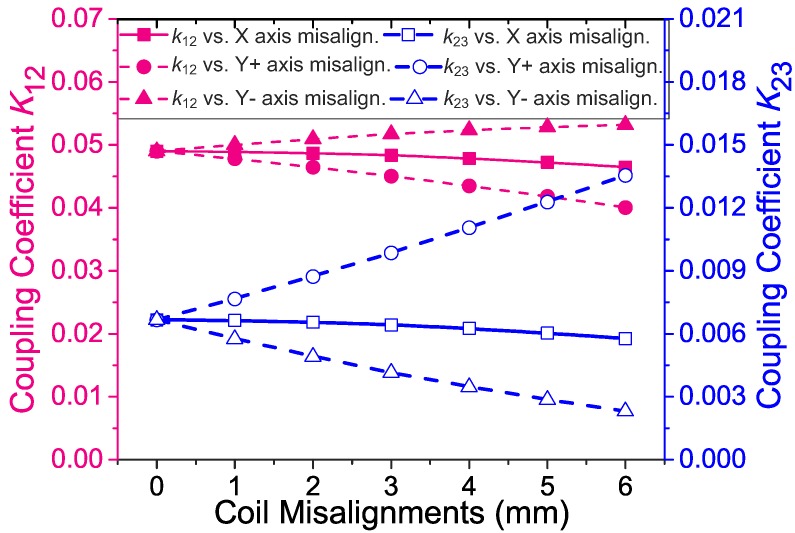
Calculated coupling coefficient $k_{12}$ and $k_{23}$ versus three different misalignments of the implant coil (‘Y+’ in the notation means misalignments in the positive direction of the Y axis, and ‘Y−’ means in the negative direction. The directions of the X axis and the Y axis are illustrated in Figure 3).

Figure 9 shows the calculated coupling coefficients ($k_{12}$ and $k_{23}$) versus the misalignments of the implant coil along the X and Y axes when the coil separation (*d*) is 20 mm. The directions of the X axis and Y axis are illustrated in Figure 3. It can be seen that both $k_{12}$ and $k_{23}$ are insensitive to the misalignments along the X axis. $k_{12}$ and $k_{23}$ are sensitive to the misalignments along the Y axis. Along with the misalignments in the Y+ axis, $k_{12}$ decreases while $k_{23}$ increases. Along with the misalignments in the Y axis, $k_{12}$ increases while $k_{23}$ decreases. This asymmetry phenomenon is due to the small offset ($d_{12}$) of the implant coil in the Y axis. In the worst case of our application, the misalignment of 6 mm in the Y axis could lead to about a $\frac{1}{3}$-times decrease of $k_{23}$. The decrease of $k_{23}$ could lead to about a −9.5 dB decrease of the received uplink signal power, which is acceptable to our uplink system because that leaves a 10-dB reliable margin for the uplink receiving as is mentioned in Section 5.

## 7. Experimental Results

### 7.1. Prototype

**Figure 10:**
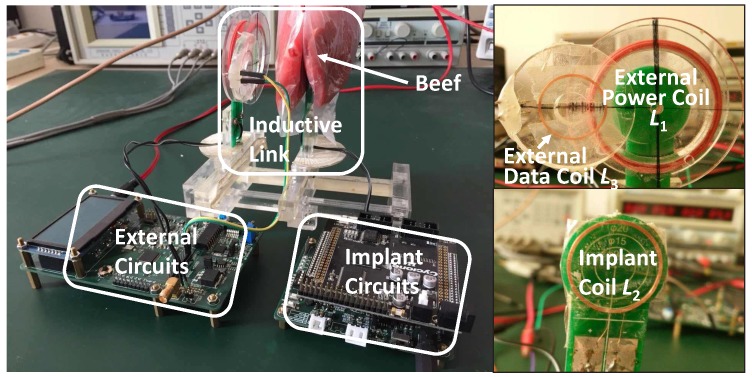
The prototype system is implemented to setup measurements of power and uplink data transmissions with beef tissue.

We implemented our prototype using discrete components and off-the-shelf chips to prove the feasibility of the future integrated circuit (IC) design, as shown in Figure 10. The coils and the inductive link circuit in the prototype are the same as those we optimized in Section 4 and Section 5. The power feeding circuit is implemented by a class-E power amplifier. The external receiving filter is implemented by a four-stage Butterworth low-pass filter. The external BPSK demodulator is digitally implemented in an MCU (STM32F207, STMicroelectronics, Geneva, Switzerland). The implant uplink power amplifier is merged in an H-bridge modulator to achieve low cost and high efficiency [24]. The implant BPSK modulator is implemented in an FPGA (Cyclone IV, Altera, San Jose, CA, USA).

### 7.2. Measurement Results

**Figure 11:**
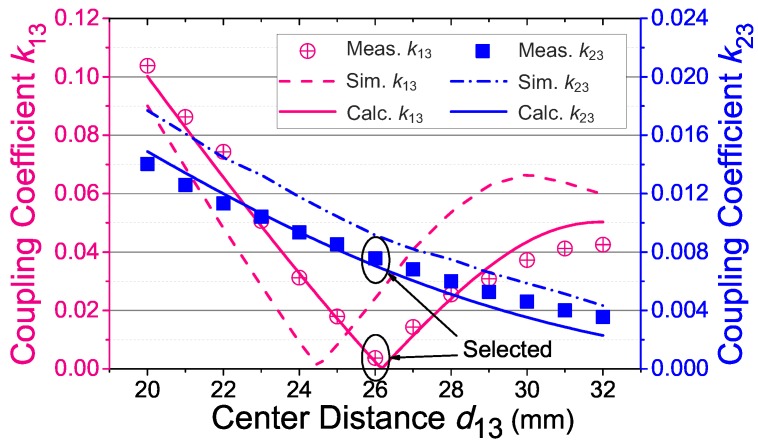
Measured (Meas.), simulated (Sim.) and calculated (Calc.) coupling coefficients ($k_{13}$, $k_{23}$) versus the center distance ($d_{13}$), when the coil separation is d=20 mm.

Figure 11 shows the measured coupling coefficients ($k_{13}$ and $k_{23}$) versus the distance ($d_{13}$). The coupling coefficients of the inductive link are measured by a network analyzer (E5071, Keysignt, Santa Rosa, CA, USA) through using the method introduced in [15]. As seen, $k_{13}$ could decrease to about zero when $d_{13}=26$ mm, which agrees with the analysis in Section 4.1. The coupling coefficient ($k_{23}$) remains at a small, but adequate value. Therefore, the power carrier interference is substantially rejected while the intensity of the uplink signal remains adequate. Figure 11 also shows the simulated and calculated $k_{13}$ and $k_{23}$ versus the center distance ($d_{13}$). It can be seen that both the simulated and the calculated coupling coefficients almost agree with the measured results with some offsets, and the calculated coupling coefficients show better agreement. It can be proven that the calculation model is in good agreement with the real coils.

**Figure 12:**
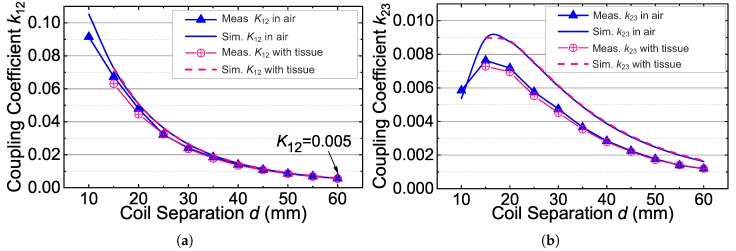
(**a**) Measured and simulated coupling coefficient $k_{12}$ versus the coil separation *d* in air and in tissue; (**b**) measured and simulated coupling coefficient $k_{23}$ versus the coil separation *d* in air and in tissue.

Figure 12a,b shows the measured $k_{12}$ and $k_{23}$ over different coil separation (*d*), as $k_{12}$ and $k_{23}$ are the key parameters of the power link and the uplink, respectively. It can be seen that the coupling coefficient $k_{12}$ is as low as 0.005, and $k_{23}$ is as low as 0.001 when the coil separation is 60 mm. In addition, Figure 12b shows that $k_{23}$ decreases when the coil separation is smaller than 15 mm, which agrees with the analysis in Section 4.2.

**Figure 13:**
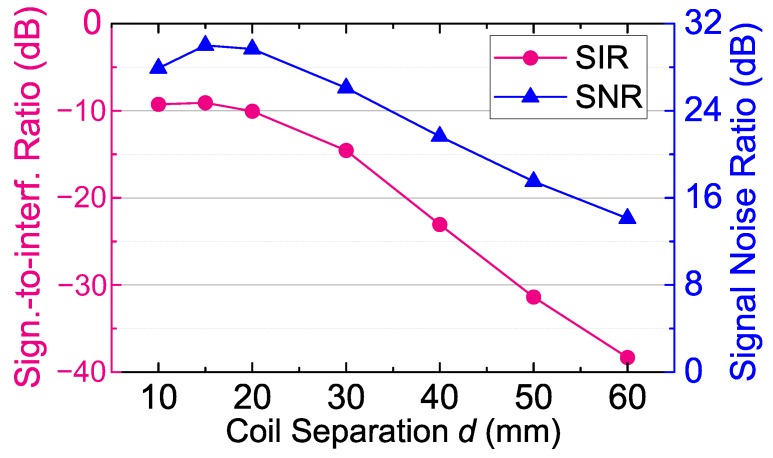
The measured signal-to-interference ratio (SIR) and the SNR of the uplink versus the coil separation *d*.

Figure 13 shows the measured SIR and SNR of the uplink over different coil separations (*d*). The SIR and SNR are measured before the uplink receiving filter by using a spectrum analyzer (DSA815, RIGOL, Beijing, China). When measuring the SIR and SNR, the power delivered to the load (PDL) is kept as constant as 5 mW by adjusting the transmitting power of the class-E power amplifier (PA). When the coil separation (*d*) is 20 mm, the SIR before the uplink receiving filter is as large as −10.07 dB. Compared with the two-coil inductive link in our previous work [11], the SIR of the magnetically-balanced inductive link has a significant improvement of 65.72 dB. When the coil separation (*d*) is as large as 60 mm (the maximum coil separation of our prototype to supply 5 mW of power to the load wirelessly), the SNR is about 14 dB, which is still adequate to achieve BPSK demodulation, and the SIR is about −38 dB, which can be improved by a fourth-order Butterworth low-pass filter. In addition, the SNR decreases when d=10 mm, since $k_{23}$ decreases. Thanks to the optimization of the skew distance $d_{12}$, this decrease is avoided in our operating range (15 mm ≤d≤ 40 mm), so it does not affect the reliability of the uplink data transmission.

To measure the bit error rate (BER), a pseudo-random data sequence is transmitted continuously at a data rate of 50 kbps in half an hour for each measurement. When measuring the BER, the PDL is kept as constant as 5 mW by adjusting the transmitting power of the class-E PA. In the experiments, no bit error occurs in half an hour when the coil separation (*d*) is within the operating range (15–40 mm); thus, the BER is below $1\times 10^{-8}$ in the operating range. When *d* is enlarged to 60 mm, the uplink is still reliable, and the measured BER is about $1\times 10^{-7}$. Although d=60 mm exceeds the expected operating range, the large distance aims to demonstrate the reliability of the proposed inductive link under ultra-weak coupling and to show the feasibility of the proposed inductive link for the future mm-sized implantable biomedical sensor devices [2,25].

**Figure 14:**
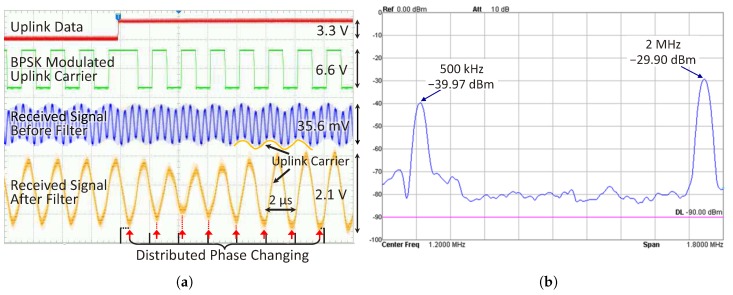
(**a**) The uplink waves captured from the prototype when the coil separation *d* = 20 mm; (**b**) the spectrum of the received uplink signal before the uplink receiving filter.

Figure 14a shows the waveforms of the transmitted and received uplink signal captured by an oscilloscope. The first wave (red) on the top is the uplink baseband data signal. The second wave (green) is the BPSK-modulated uplink carrier, which is measured at the output of the implant transmitter (before being merged with the implant coil through $R_{\mathrm{isolate}+}$ and $R_{\mathrm{isolate}-}$). The third wave (blue) is the received signal before the uplink receiving filter, which is measured after the pre-amplifier of the uplink receiving filter. In order to show that there are two major components in the received signal, Figure 14b shows the spectrum of the received uplink signal. It can be clearly seen that one component is the 2-MHz power carrier interference, and the other is the 500-kHz uplink carrier. The last wave (orange) on the bottom is the received signal after the filter, which is measured at the output of the uplink receiving filter. After filtering, the uplink carrier is clearly amplified, and the power carrier is completely blocked. The phase changing of the received uplink carrier is distributed over about eight carrier cycles. Through observing the red arrows, which point to the valleys of the received signal, it can be seen that the phase of the received signal slowly changes. As the horizontal time base is 2 μs per division, which is equal to the period of the uplink carrier, each vertical grid line can be viewed as the reference of the carrier phase for each cycle. The relative position of the valleys in each cycle moves about half a grid comparing to the vertical grid line during the eight cycles, which corresponds to the 180° phase changing. The phase changing is not immediate like that in transmitted signal due to the narrow band link formed by the external data coil $L_{2}$ and its tuning capacitor $C_{2}$. The amplitude of the received signal decreases slightly also due to the phase changing in the narrow band link.

**Figure 15:**
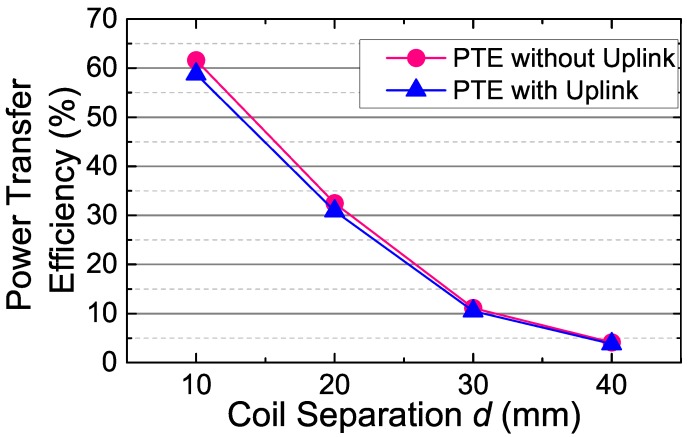
The power transfer efficiency versus coil separation *d* with or without connecting the uplink circuits, which includes the isolation resistors and the uplink transmitter. PTE, power transfer efficiency.

To evaluate the impacts of the uplink data transmission on the power link, we measured the PTE of the proposed inductive link. The PTE is measured by a network analyzer (Keysignt E5071) through using the method in [26]. To provide a comparison baseline, the PTE of the pure power inductive link without connection of the uplink transmitter was also measured. The measured results are shown in Figure 15. Although the power link and uplink share the same implant coil, the PTE still maintains at a fairly high level with negligible loss (only ∼5% loss of PTE). This is benefited from the high impedance resistors $R_{\mathrm{isolate}-}$ and $R_{\mathrm{isolate}+}$, which isolate the impacts of the uplink data transmission on the power harvesting. In addition, $V_{\mathrm{load}}$ were measured when the implant uplink transmitter is on, to estimate the ripples of $V_{\mathrm{load}}$ caused by the uplink data transmission. The uplink data transmission caused ∼5% ripple on $V_{\mathrm{load}}$ before regulation (according to the oscilloscope downloaded data). That is, the uplink data transmission only causes ∼5% variation of the power carrier amplitude on the load.

### 7.3. Tissue and Safety

**Figure 16:**
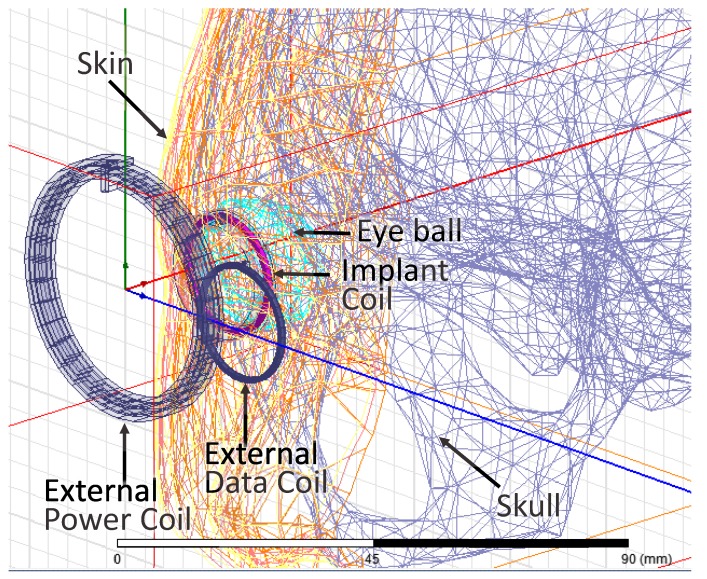
The magnetically-balanced inductive link is simulated with the human head model in ANSYS HFSS electromagnetic simulation suite.

In order to assess the impacts of tissue on the inductive link, the implant coil is wrapped in 10 mm-thick beef tissue as seen in Figure 10. According to the work in [27], the permittivity and the conductivity of the beef muscle tissue are close to those of the human muscle tissue when the frequency is in the level of MHz. However, the tissues in the eyeball need to be further studied, because our implant coil is designed to be implanted around the eyeball. Thus, we further place the inductive link in the specific true-CAD human head model and simulate them in the ANSYS HFSS electromagnetic simulation suite as seen in Figure 16. There are tissues of skin, fat, muscle, eyeball and skull in the human head model. The true-CAD human head model is obtained from NEVA Electromagnetics [28]. The permittivity and the conductivity of the tissues are obtained from [29]. In the simulation, the implant coil is wrapped in silica gel and surrounds the periphery of the cornea hidden behind the eyelid.

Figure 12 shows the simulated and measured coupling coefficients ($k_{12}$ and $k_{23}$) over different coil separations (*d*). The simulated coupling coefficients are in good agreement with the measured results. Figure 12 also shows that the measured coupling coefficients when the implant coil is wrapped in the beef tissue and the simulated coupling coefficients when the implant coil is placed in the human body model. Both of the measured results and the simulated results present that the coupling coefficients when the inductive link is in the tissue are almost the same as those when the inductive link is in air. The tissues scarcely affect the coupling coefficients of this link, because the absorption of the tissues towards the low frequency magnetic field is negligible. In addition, the tissue could increase the parasitic capacitance of the implant coil [30]. This may lower the PTE due to the detuning. Benefiting from the selection of the low frequency (2 MHz) power carrier, the detuning caused by the tissue is minor and negligible in our inductive link.

In order to assess the radio frequency (RF) exposure safety on the human body, we used the ANSYS HFSS electromagnetic simulation suite to calculate the specific absorption rate (SAR). The simulation results show that the maximal local SAR in tissues is about three orders lower than the basic restriction of 2 W/kg [31].

In addition, there could be temperature increase in the human body due to the power consumed by the implant coil and implant circuits. According to the comprehensive thermal analysis on an intraocular implant [32], our device with 5-mW power consumption may induce the maximum temperature increase of approximately 0.4 °C on the surface of the device. The temperature increase is below the guideline 1 °C in [31].

### 7.4. Comparison with the State-of-the-Art

A comparison of the proposed inductive link system with other published works is shown in Table 2. The comparison is specific to the designs that use the inductive link for the simultaneous power and uplink data transmissions, where the power link could highly limit the performance of the uplink.

Compared with the conventional LSK method [5,33], the new LSK methods [9] and the PPSK method [10], our work achieves reliable uplink data transmission under a much lower coupling coefficient and larger coil separation. At the same time, our uplink coexists better with the WPT. When the wireless power of our work is continuously transferred to the implant, the uplink data are reliably transmitted to the external coil with negligible impacts on WPT (only 5.1% loss of PTE and 5% ripple on $V_{\mathrm{load}}$).

Compared with the multiple coil pair method [12], our work also achieves the simultaneous power and uplink data transmissions under a lower coupling coefficient and larger coil separation. Furthermore, our work only uses one implant coil for both harvesting wireless power and transmitting uplink data, which will be feasible for some specific applications that cannot implant multiple coils.

The work in [11] is our previous dual-carrier uplink system, but using the conventional two-coil inductive link. It can transfer uplink data and wireless power reliably simultaneously through the same coil pair under weak coupling. However, the uplink in the previous work suffers a severe power carrier interference (SIR = −75.79 dB), which highly challenges the circuit design of the receiver and highly limits the performance of the uplink. Compared with [11], the proposed magnetically-balanced inductive link achieves a 65.72-dB improvement of the SIR. This is a significant improvement for the weak uplink signal receiving, which remarkably alleviates the challenge of receiving the weak uplink data signal under strong power carrier interference. In addition, the uplink data transmission causes only 5.1% loss of the PTE in the proposed inductive link, which is much less than that in the previous works. Therefore, compared with the conventional two-coil inductive link, the magnetically-balanced inductive link achieves significant improvement of SIR and less loss of PTE.

## 8. Conclusions

This paper proposes a new magnetically-balanced inductive link for the simultaneous power and uplink data transmissions under ultra-weak coupling. An extra external coil is specially added for the uplink receiving. The power carrier interference is minimized to approach zero by balanced canceling of the magnetic field of the power carrier. We optimize the coil parameters to achieve higher SIR based on the modeling of the mutual inductance. We also optimize the circuit parameter to minimize the impact of the uplink data transmission on the PTE. We use our intraocular sensor device as an application example to present the modeling and the optimization procedure in detail. The prototype achieves as high as a 65.72-dB improvement of the SIR compared with the conventional two-coil inductive link. Benefiting from this substantial improvement of SIR, the inductive link achieves a 1 × 10${}^{-7}$ BER uplink data transmission even though the coupling coefficient is as low as 0.005, and at the same time, the uplink coexists well with the power link with only ∼5% PTE loss and ∼5% voltage ripple on the implant load. This magnetically-balanced inductive link could be useful for small-sized biomedical sensor devices, which require transmitting data and power simultaneously under ultra-weak coupling.

## Figures and Tables

**Table 1:** Parameters of the coils.

Parameter	$L_{1}$	$L_{2}$	$L_{3}$
Coil Wire Type	Litz wire with 11 strands of 30 AWG	Enameled copper	Enameled copper
Wire Diameter	0.8 mm	0.06 mm	0.06 mm
Coil Radius	20 mm	10 mm	8 mm
Coil Thickness	4.5 mm	0.12 mm	0.12 mm
Number of Turns	$N_{1}$ = 30	$N_{2}$ = 35	$N_{3}$ = 25
Self Resonated Frequency	7 MHz	6 MHz	11 MHz
Coil Inductance (2 MHz/500 kHz)	68.2/63.0 μH	72.9/63.6 μH	25.9/24.9 μH
Equivalent Series Resistance (2 MHz/500 kHz)	8.5/1.9 Ω	25.9/16.2 Ω	10.2/7.9 Ω
Quality Factor (2 MHz/500 kHz)	101/103	35.4/12.3	31.9/9.9

The coil inductance, equivalent series resistance and quality factor are measured by a precision impedance analyzer (E4990A, Keysignt, Santa Rosa, CA, USA).

**Table 2:** Performance comparison. PDL, power delivered to the load; LSK, load-shift keying; PPSK, passive phase shift keying; OQPSK, offset quadrature phase shift keying; WPT, wireless power transmission; Ext., external; Im., Implant.

References	[5]	[33]	[9]	[12]	[10]	[11]	This Work
Ext./Im. Coil No.	1/1	2/1	2/1	3/3	1/1	1/1	**2/1**
Ext./Im. Coil Diameter (mm)	60/20	40,25/9.5	56,22/22	24,12,12/24,12,12	25/16	40/20	40,16/20
**Wireless Power Transfer**							
Power Carrier (Hz)	700 × 10${}^{3}$	13.56 × 10${}^{6}$	1.1/1.53 × 10${}^{6}$	1 × 10${}^{6}$	13.56 × 10${}^{6}$	2 × 10${}^{6}$	2 × 10${}^{6}$
Typical PDL (mW)	50	102	60	1.5∼12	≤100	10	5
Typical PTE	36%	50%	36%	61%	58% (k = 0.1)	59% (k = 0.1)	59% (k = 0.09)
**Uplink Data Transmission**							
Modulation	LSK	LSK	Double Carrier LSK	OQPSK	PPSK	BPSK	BPSK
Uplink Carrier (Hz)	700 × 10${}^{3}$	13.56 × 10${}^{6}$	1.1/1.53 × 10${}^{6}$	13.56 × 10${}^{6}$	13.56 × 10${}^{6}$	125 × 10${}^{3}$	500 × 10${}^{3}$
Data Rate (bps)	19.2 × 10${}^{3}$	56.5 × 10${}^{3}$	10 × 10${}^{3}$	4.16 × 10${}^{6}$	1.35 × 10${}^{6}$	10 × 10${}^{3}$	50 × 10${}^{3}$
SIR (dB)	NA	NA	NA	NA	NA	−75.79	−10.07
Tx Power Cost (mW)	NA	NA	NA	NA	NA	0.6	**0.2**
Max. Distance (mm)	30	20	11	5	15	50	**60**
Coupling Coefficient ${}^{a}$	0.045	0.04 ${}^{b}$	0.23	0.23	0.055	0.008	**0.005**
BER	NA	NA	NA	≤$2\times 10^{-6}$	≤$1\times 10^{-5}$	≤$1\times 10^{-7}$	**≤$1\times 10^{-7}$**
**The Impacts of Uplink Data Transmission on WPT**							
PTE Relative Loss	>50%	NA	19%	NA	20% (k = 0.05)	15% (k = 0.05)	**5.1% (k = 0.05)**
$V_{\mathrm{load}}$ Ripple	NA	NA	15%	NA	100%${}^{c}$	∼5%	**∼5%**

${}^{a}$ If there are multiple coupling coefficients for a system, only the largest one is used for the comparison. ${}^{b}$ Simulated by using ANSYS HFSS from the coil parameters. ${}^{c}$ The values of ripple are read from the oscilloscope waves of the load voltage in the references.

**Figure A1:**
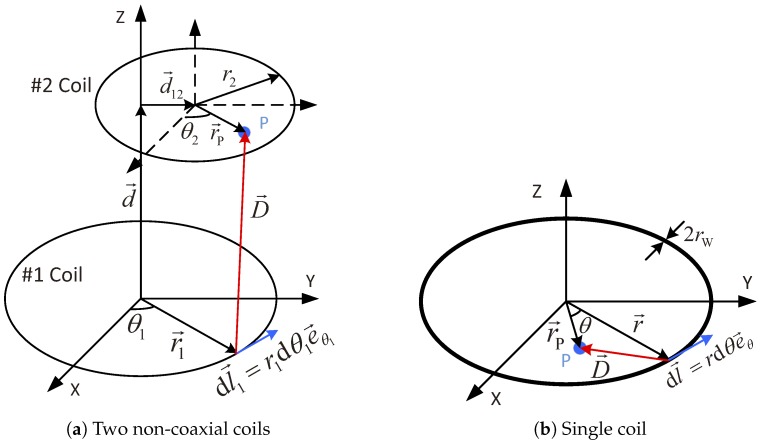
(**a**) The model for calculating mutual inductance of two non-coaxial coils with a single loop; (**b**) the model for calculating the self-inductance and self of a coil with a single loop.

**Figure A2:**
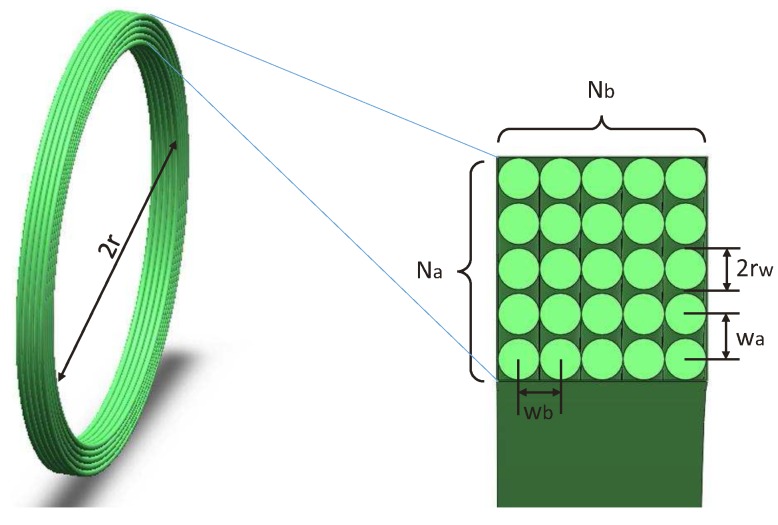
Geometric arrangement and notation of the coil with multiple turns.

## Appendix A. Modeling of Self- and Mutual Inductance

The self- and mutual inductances of the coils need to be carefully modeled for the coil parameter optimizations. Figure A1a shows the model for calculating the mutual inductance between two circular filamentary coils with a single loop (Coil #1 and Coil #2). The word filamentary means that the coils satisfy the condition $\frac{r_{w}}{r}\ll 1$, where $r_{w}$ is the radius of the wire and *r* is the radius of the coil. Figure A1b shows the model for calculating the self-inductance of a circular filamentary coil with a single loop. According to Faraday’s law of induction, we have Equations (A1) and (A2), where Ψ is the magnetic flux linkage of the coil induced by itself and $\Psi_{12}$ is the magnetic flux linkage of Coil #2 induced by Coil #1. As seen, the inductance can be calculated through dividing the magnetic flux linkage by the current. In the physics definition, the magnetic flux linkage is an integration of the magnetic field ($\vec{B}$) covering a surface, and $\vec{B}$ can be derived by using the Biot–Savart law.

(A1) $$V=\frac{d\Psi }{dt}=L\frac{dI}{dt}\Rightarrow \Psi =LI,$$

(A2) $$V_{2}=\frac{d\Psi_{12}}{dt}=M_{12}\frac{dI_{2}}{dt}\Rightarrow \Psi_{12}=M_{12}I_{2}.$$

For the case of Figure A1a, the magnetic field $\vec{B}$ at each point *P* is integrated covering the entire surface of Coil #2 to calculate the magnetic flux linkage through Coil #2. As the magnetic field perpendicular to the surface contributes to the magnetic flux, the magnetic field ${\vec{B}}_{z}$ in the Z axial direction is integrated. The brief calculation procedure of the magnetic flux linkage ($\Psi_{12}$) is listed in Equations (A3)–(A8). Then, the mutual inductance $M_{12}$ between Coil #1 and Coil #2 is derived in Equation (A9).

(A3) $$\begin{array}{cc} \vec{D} & =-{\vec{r}}_{1}+\vec{d}+{\vec{d}}_{12}+{\vec{r}}_{p}=\left(r_{p}\cos\theta_{2}-r_{1}\cos\theta_{1}\right){\vec{e}}_{x}+\left(r_{p}\sin\theta_{2}-r_{1}\sin\theta_{1}+d_{12}\right){\vec{e}}_{y}+d{\vec{e}}_{z}, \end{array}$$

(A4) $$\begin{array}{cc} d{\vec{l}}_{1} & =r_{1}d\theta_{1}{\vec{e}}_{\theta_{1}}=r_{1}\left(-\sin\theta_{1}{\vec{e}}_{x}+\cos\theta_{1}{\vec{e}}_{y}\right)d\theta_{1}, \end{array}$$

(A5) $$\begin{array}{cc} d\vec{B} & =\frac{\mu_{0}}{4\pi }\cdot \frac{I_{1}d{\vec{l}}_{1}\times \vec{D}}{|\vec{D}{|}^{3}}, \end{array}$$

(A6) $$\begin{array}{cc} d{\vec{B}}_{z} & =\frac{\mu_{0}Ir_{1}}{4\pi }\cdot \frac{r_{1}-r_{p}\cos\left(\theta_{1}-\theta_{2}\right)-d_{12}\sin\theta_{1}}{|\vec{D}{|}^{3}}{\vec{e}}_{z}d\theta_{1}, \end{array}$$

(A7) $$\begin{array}{cc} {\vec{B}}_{z} & =\oint d{\vec{B}}_{z}=\int_{0}^{2\pi }\frac{\mu_{0}Ir_{1}}{4\pi }\cdot \frac{r_{1}-r_{p}\cos\left(\theta_{1}-\theta_{2}\right)-d_{12}\sin\theta_{1}}{|\vec{D}{|}^{3}}{\vec{e}}_{z}d\theta_{1}, \end{array}$$

(A8) $$\begin{array}{cc} \Psi_{12} & =\int \;\;\;\;\;\int \;◯{\vec{B}}_{z}{\vec{e}}_{z}ds=\int_{0}^{2\pi }\int_{0}^{r_{2}}\left|{\vec{B}}_{z}\right|r_{p}dr_{p}d\theta_{2}, \end{array}$$

(A9) $$\begin{array}{cc} \;M_{12} & =\frac{\Psi_{12}}{I}=\int_{0}^{2\pi }\int_{0}^{r_{2}}\int_{0}^{2\pi }\frac{\mu_{0}r_{1}r_{p}}{4\pi }\cdot \frac{\left(r_{1}-r_{p}\cos\left(\theta_{1}-\theta_{2}\right)-d_{12}\sin\theta_{1}\right)d\theta_{1}dr_{p}d\theta_{2}}{{\left({\left(r_{p}\cos\theta_{2}-r_{1}\cos\theta_{1}\right)}^{2}+{\left(r_{p}\sin\theta_{2}-r_{1}\sin\theta_{1}+d_{12}\right)}^{2}+d^{2}\right)}^{\frac{3}{2}}}. \end{array}$$

Equation (A9) can be simplified and rewritten by a single integral as in Equations (A10) and (A11) according to [34,35,36], where $J_{0}\left(x\right)$/$J_{1}\left(x\right)$ is the zero/first order Bessel function of the first kind, K(s)/E(s) is the complete elliptic integral of the first/second kind, $h\equiv \sqrt{{r_{1}}^{2}+{d_{12}}^{2}-2r_{1}d_{12}\cos\phi }$ and $s\equiv \sqrt{4r_{2}h/\left({\left(r_{2}+h\right)}^{2}+d^{2}\right)}$. Equations (A9)–(A11) are equivalent and can be calculated by using the integral3/integral function in MATLAB.

(A10) $$\begin{array}{cc} M\left(r_{1},r_{2},d_{12},d\right) & =\mu_{0}\pi \sqrt{r_{1}r_{2}}\int_{0}^{\infty }J_{1}\left(x\sqrt{\frac{r_{1}}{r_{2}}}\right)J_{1}\left(x\sqrt{\frac{r_{2}}{r_{1}}}\right)J_{0}\left(x\frac{d_{12}}{\sqrt{r_{1}r_{2}}}\right)\exp\left(-x\frac{d}{\sqrt{r_{1}r_{2}}}\right)dx \end{array}$$

(A11) $$\begin{array}{c} =\frac{\mu_{0}r_{1}r_{2}}{\pi }\int_{0}^{\pi }\frac{r_{1}-d_{12}\cos\phi }{h\sqrt{r_{2}h}}\left(\left(\frac{2}{s}-s\right)K\left(s\right)-\frac{2}{s}E\left(s\right)\right)d\phi . \end{array}$$

The calculation of self-inductance in Figure A1b is to integrate the magnetic flux linkage induced by itself. As the derivation procedure is similar to that of the mutual inductance, only the final derived formula is shown in Equation (A12). According to [37], the self-inductance of such a loop coil can be approximated by Equation (A13). Equations (A12) and (A13) are in good agreement with the condition $\frac{r_{w}}{r}\ll 1$. Note that $r_{w}$ cannot be regarded as zero although $\frac{r_{w}}{r}\ll 1$. If $r_{w}$ were zero, the self-inductance would be infinite. The wire radius ($r_{w}$) is important to the calculation of the self-inductance, and it should be the exact radius of the conductor.

(A12) $$\begin{array}{cc} L\left(r,r_{w}\right) & =\frac{\mu_{0}r}{2}\int_{0}^{r-r_{w}}\int_{0}^{2\pi }\frac{r_{p}\left(r-r_{p}\cos\theta \right)}{{\left(r^{2}+{r_{p}}^{2}-2rr_{p}\cos\theta \right)}^{\frac{3}{2}}}d\theta dr_{p} \end{array}$$

(A13) $$\begin{array}{c} \approx \mu_{0}r\left(\ln\left(\frac{8r}{r_{w}}\right)-2\right). \end{array}$$

If the condition $\frac{r_{w}}{r}\ll 1$ is not satisfied for some coils, the concept of the geometric mean distance (GMD) between coils [38] can be used. By replacing the separation distance (*d*) with the GMD, Equations (A9) and (A12) can still be valid.

In the inductive link, the coils are wound turn by turn and layer by layer as shown in Figure A2. Figure A2 shows a coil with $N_{b}$ turns per layer and $N_{a}$ layers. The distance between adjacent turns is $w_{b}$, and the distance between adjacent layers is $w_{a}$.

For the coil with multiple turns, the total self-inductance can be calculated as Equation (A14), where $\delta_{\left(a_{i},a_{j}\right),\left(b_{i},b_{j}\right)}=1$ if $a_{i}=a_{j}$ and $b_{i}=b_{j}$. The total self-inductance consists of two parts, which are the self-inductance of loops and the mutual inductance between loops. Note that if two loops are coaxial in the same plane ($a_{i}\neq a_{j}$ and $b_{i}=b_{j}$), the equations of mutual inductance (Equations (A10) and (A11)) have a poor calculation accuracy in MATLAB due to the divergence of their integrals. By reviewing the formula derivation procedures of mutual inductance and self-inductance for a single loop, we realize that the magnetic flux integration process of the self-inductance is the same as that of the mutual inductance between loops in the same plane. If the layer distance between the two loops is regarded as the wire radius of the single loop, the equation of mutual inductance (Equation (A9)) and the equation of self-inductance (Equation (A12)) can be equivalent. The equation of self-inductance has better integral convergence than that of mutual inductance on the condition of d=0 mm. Thus, it is suggested to use the equation of self-inductance to calculate the mutual inductance when the two loops are coaxial in the same plane, as shown in Equation (A15).

(A14) $$\begin{array}{c} L_{\mathrm{total}}\left(r,r_{w},N_{a},N_{b},w_{a},w_{b}\right)=\sum_{a_{i}=1}^{Na}\sum_{b_{i}=1}^{N_{b}}L\left(r+w_{a}\left(a_{i}-1\right),r_{w}\right)\\ +\sum_{a_{i}=1}^{Na}\sum_{b_{i}=1}^{N_{b}}\sum_{a_{j}=1}^{Na}\sum_{b_{j}=1}^{N_{b}}M\left(r+w_{a}\left(a_{i}-1\right),r+w_{a}\left(a_{j}-1\right),0,w_{b}\left|b_{i}-b_{j}\right|\right)\left(1-\delta_{\left(a_{i},a_{j}\right),\left(b_{i},b_{j}\right)}\right). \end{array}$$

(A15) $$M\left(r+w_{a}\left(a_{i}-1\right),r+w_{a}\left(a_{j}-1\right),0,0\right)=L\left(r+w_{a}\left(\max\left(a_{i},a_{j}\right)-1\right),w_{a}\left|a_{i}-a_{j}\right|+r_{w}\right).$$

For two non-coaxial coils with multiple turns, the total mutual inductance can be calculated as Equation (A16), where $r_{1}$, $N_{a1}$, $N_{b1}$, $w_{a1}$ and $w_{b1}$ are the radius, the number of layers, the number of turns per layer, the distance between adjacent layers and the distance between adjacent turns of Coil #1, respectively, and $r_{2}$, $N_{a2}$, $N_{b2}$, $w_{a2}$ and $w_{b2}$ are those of Coil #2, while $d_{12}$ and *d* stand for the offset distance and the separation distance between the two coils.

(A16) $$\begin{array}{c} M_{total}\left(r_{1},r_{2},d_{12},d,N_{a1},N_{b1},w_{a1},w_{b1},N_{a2},N_{b2},w_{a2},w_{b2}\right)=\\ \sum_{a_{i}=1}^{N_{a1}}\sum_{b_{i}=1}^{N_{b1}}\sum_{a_{j}=1}^{N_{a2}}\sum_{b_{j}=1}^{N_{b2}}M\left(r_{1}+w_{a1}\left(a_{i}-1\right),r_{2}+w_{a2}\left(a_{j}-1\right),d_{12},d+w_{b1}\left(b_{i}-1\right)+w_{b2}\left(b_{j}-1\right)\right). \end{array}$$

Finally, the coupling coefficient between the two coils with multiple turns can be calculated by using the total self-inductance and mutual inductance, as shown in Equation (A17).

(A17) $$k=\frac{M_{\mathrm{total}}}{\sqrt{L_{\mathrm{total}1}L_{\mathrm{total}2}}}.$$
